# Phosphorus removal from irrigation return flow using an iron oxide filter and denitrifying pine bark bioreactor treatment train

**DOI:** 10.1007/s11356-024-35641-4

**Published:** 2024-12-04

**Authors:** Jack L. Dekle, William H. J. Strosnider, Sarah A. White

**Affiliations:** 1 Environmental Toxicology Graduate Program, Clemson University, 509 Westinghouse Rd., Pendleton, SC 29670 USA; 2 Baruch Marine Field Laboratory, University of South Carolina, Georgetown, SC 29442 USA; 3 Department of Plant and Environmental Sciences, E-143 Poole Agricultural Center, Clemson University, Clemson, SC 29634 USA; 4 Present Address: Brown and Caldwell, Atlanta, GA 30328 USA

**Keywords:** Sorption capacity, Water treatment, Reclaimed iron oxide, Treatment order, Nutrient remediation, Water reuse

## Abstract

Development of low-cost aqueous P removal methods is imperative for water resource protection. This study assessed the contribution of an iron oxide (FeOx) filter for P sorption paired with a denitrifying pine bark bioreactor, quantifying the effect of treatment order on P removal. FeOx filters were placed upstream (order 1) or downstream (order 2) of pine bark bioreactors receiving a continuous flow of simulated irrigation return flow after constructed floating wetland treatment. The FeOx filters removed 0.095 ± 0.01 g P·m^−3^·d^−1^ and 0.21 ± 0.01 g P·m^−3^·d^−1^ in the spring and fall, respectively. P concentration was reduced from 5.08 to 3.8 mg·L^−1^ and from 6.72 to 4.5 mg·L^−1^ in the spring and fall experiments, respectively. The FeOx substrate sorbed 1.49 ± 0.08 mg P·g FeOx^−1^ in spring and 3.18 ± 0.2 mg P·g FeOx^−1^ fall experiments. P sorption varied by season due to differences in the load presented to the FeOx filters. Reclaimed FeOx substrates were viable P removal filters, especially during cooler months when the nutrient uptake capacity of constructed floating wetland plants was limited. Overall, findings indicate that FeOx filters can be used as a substrate for P sorption in conjunction with constructed floating wetlands or other plant-based treatment technologies that can be limited by seasonality.

## Introduction

The environmental relevance of legacy phosphorus (P) contamination has led to water quality monitoring and remediation efforts, as P is a limiting nutrient in eutrophication and primary productivity of algae in freshwaters (Correll 1999). Agriculture and specialty crop operations are nonpoint source contributors of excess P in many watersheds (Sharpley 2016). Agricultural, stormwater, and industrial wastewater treatment operations apply various nutrient remediation strategies such as edge-of-field buffers and constructed wetlands to reduce nonpoint nitrogen (N) and P runoff and treat irrigation return flow for reuse (Dabney et al. 2006). A variety of substrates such as zeolite, steel slag, calcined clay, and iron (Fe) oxides have been used as low-cost P adsorbents to treat wastewater effluent, activated sludge, and agricultural runoff (Andrés et al. 2018, Kang et al. 2003; Lalley et al. 2016; Penn et al. 2016).

Sorption of P onto substrate surfaces reduces dissolved P concentrations in aquatic ecosystems. Fe-based media have been used in constructed wetlands and as a modification to constructed floating wetlands (CFWs) to supplement plant P removal (Pant et al. 2001; Wang et al. 2018). Christianson et al. (2017) reported greater P removal capacity in Fe-based P-filters than calcium-based steel slag filters. Fe-based media, particularly FeOx from coal mine drainage residuals, are a more sustainable substrate filter option because of the potential for reuse. The surface area and surface reactivity of FeOx make FeOx an ideal substrate for P removal via adsorption. This removal mechanism consists of P adsorption on the generally positively charged surface of FeOx substrates through the following reaction: Fe-OH + H_2_PO_4_^−^ → Fe–O-H_2_PO_3_ + ^−^OH. In addition to removing P from irrigation return flow via adsorption, FeOx can also be reused as a soil amendment, where P-saturated sites will slowly desorb P for plant uptake (White et al. 2021). Given that FeOx is a waste product of mine drainage treatment, recovery of FeOx for P removal presents a sustainable management approach. FeOx may be used in a circular P economy through utilization of a waste product for multiple applications in water quality protection and agriculture.

Denitrifying bioreactors are a low-cost option for N removal where woody substrates serve as a carbon source to facilitate microbial denitrification of nitrate (NO_3_^−^) into nitrogen gas (N_2_) (Lopez-Ponnada et al. 2017). Woodchip substrates in denitrifying bioreactors have a lifespan of up to 10 years, and recent studies have used alternatives such as pine bark and corn cob as bioreactor substrates (Lepine et al. 2018; Malá et al. 2017; Yu et al. 2019). However, bioreactor design is critical to maximizing N removal, as excessively long hydraulic retention times (HRTs) can cause incomplete denitrification, low-quality effluent, and pollution swapping—where reduction of N results in an increase of P and vice versa. Poor bioreactor design or management can lead to high concentrations of dissolved organic carbon and highly reducing conditions within bioreactor effluent, influencing P solubility and reducing P adsorption to FeOx (Weng et al. 2012).

Research has explored the potential of dual nutrient (N and P) removal by addition of P-filter media to denitrifying woodchip bioreactors, but P sorption is limited by preferential flow paths, differences in media lifespan, and feasibility of field scale application (Bock et al. 2016; Christianson et al. 2017; Gottschall et al. 2016). Two-phase treatment trains have been implemented by placing N and P removal technologies in sequence to improve the respective nutrient removal capacities. Goodwin et al. (2015) reported optimized P removal when steel turnings (dominated by zero-valent Fe) from an industrial facility were placed downstream from a woodchip bioreactor, where the opposite order optimized N removal. Christianson et al. (2017) also reported optimized P removal rates when P-filters were placed downstream from woodchip bioreactors. However, upstream P-filter placement was preferred when woodchip bioreactor effluent was highly reduced due to long HRTs.

Though some research has been conducted on the use of treatment trains, few published studies have explored this pairing method with P filters continuously receiving water with a high P concentration. Thus, the goals of this study were to (1) quantify the P remediation contribution of an FeOx filter receiving an elevated P load and (2) compare the P removal efficiency of an FeOx filter when placed upstream or downstream of a denitrifying pine bark bioreactor. The results of this study will help potential users assess the design and lifespan of FeOx-based sorption filters for treating P-enriched waters from agricultural, municipal, stormwater, and industrial sources.

## Methodology

### Supplemental nutrient removal treatments

This experiment was set up and monitored at the Water Treatment Technology Laboratory at the South Carolina Water Resources Center in Pendleton, SC. Two replications of this study took place over a spring season, from February 20 to July 2, 2020 (133 days, 19 weeks), and a fall season, from October 1, 2020, to March 14, 2021 (164 days, 23 weeks). Supplemental nutrient removal treatments were used in conjunction with a mesocosm scale CFW experiment. The supplemental treatments received effluent from preceding CFW mesocosms using the experimental setup previously described in Dekle et al. (2024).

**Fig. 1:**
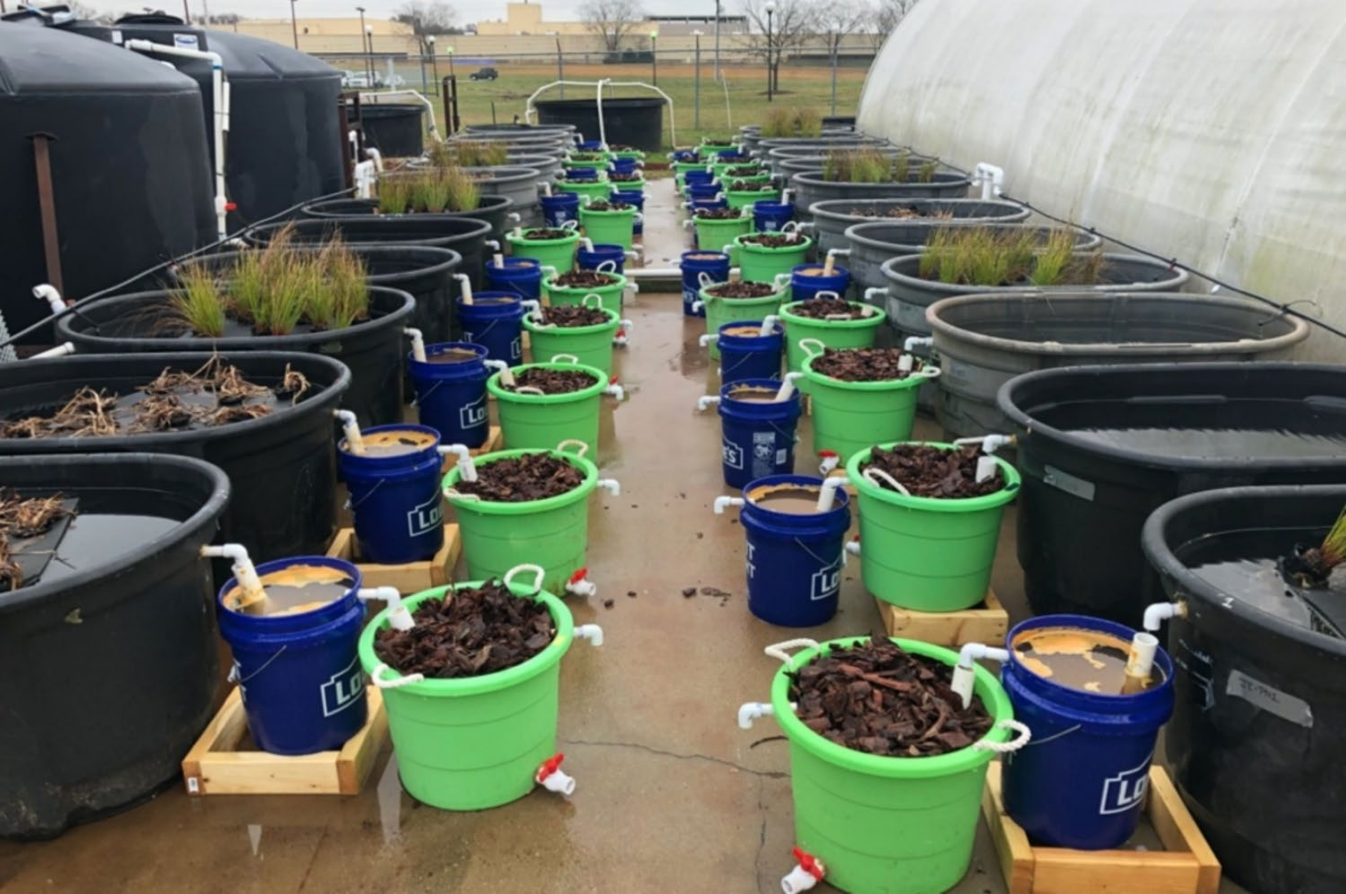
Full experimental setup with supplemental treatment trains in alternating orders (*n* = 12 for each treatment order sequence)

**Fig. 2:**
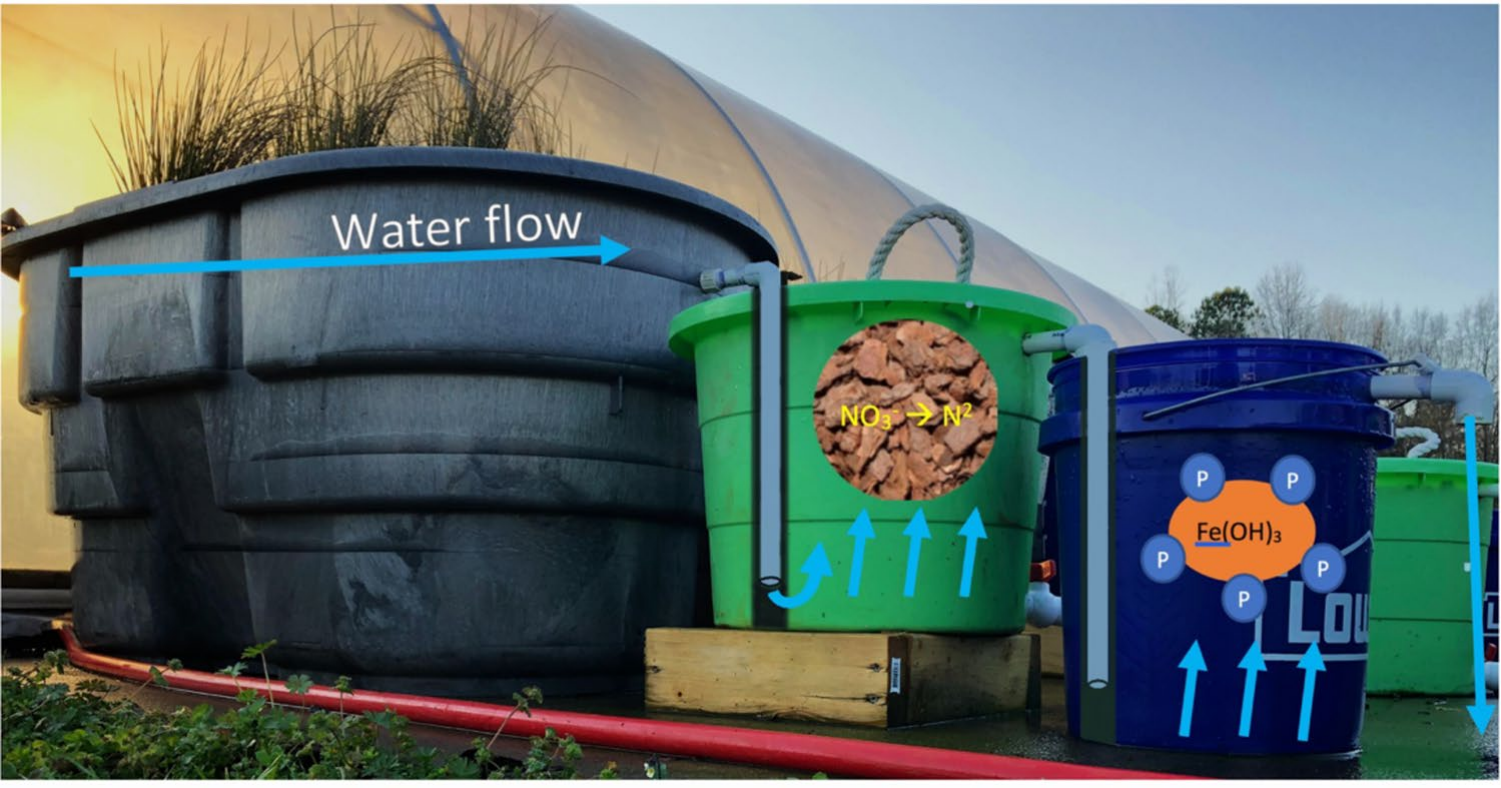
Diagram of simulated irrigation water flow through CFW mesocosms and into supplemental treatment train. Water was plumbed to the bottom of each downstream treatment to allow for maximum substrate contact and complete mixing

Two supplemental nutrient removal treatments followed each CFW treatment: (1) a FeOx filter and (2) a pine bark bioreactor. Supplemental treatments were arranged in alternating order (FeOx ➔ Bioreactor, Bioreactor ➔ FeOx), with four replicates of each arrangement per plant treatment (Fig. 1). The order 1 treatment sequence consisted of an FeOx filter followed by a pine-bark bioreactor, and the order 2 sequence consisted of a pine-bark bioreactor followed by an FeOx filter. The alternating treatment orders were tested to determine the nutrient removal contribution of each treatment technology and to assess the dynamics of nutrient removal mechanisms when placed in sequence. Additionally, the combined CFW and supplemental treatment sequence was assessed to quantify the P removal contribution of the treatment system as a whole. Water from the mesocosms was plumbed through a PVC pipe for release at the bottom of the following supplemental treatment to maximize substrate surface area contact (Fig. 2).

Water samples were collected for nutrient analysis from an outflow valve of each treatment every 7 days to quantify the nutrient remediation contribution of each respective supplemental treatment. Water samples were collected into 15 mL vials and immediately stored at 4 °C without filtration or acidification. Water samples collected from each treatment were filtered and acidified prior to analysis using inductively coupled plasma optical emission spectroscopy (ICP- OES, iCAP 6500, Thermo Scientific, Waltham, MA) for P, K, Ca, Mg, Zn, Cu, Mn, Mo, Ni, Fe, S, Na, B, and Al. All ICP analyses had calibration standards rerun at the midpoint and end of each analytical run to produce repeated and reliable analyses of samples. Additionally, water quality parameters, including pH, dissolved oxygen (DO), oxidation–reduction potential (ORP), and temperature, were measured (Table 1) in the FeOx filter containers every 7 days using a calibrated multi-parameter handheld sonde, with individual sensors for each parameter (YSI ProODO, Yellow Springs, OH; Hanna Instruments, Woonsocket, RI). Water quality measurements were recorded at 17 cm depth after the sensors had fully stabilized. Sondes were cleaned between each container, and water quality measurements were recorded between 1100 and 1500 on the sampling days.

**Table 1:** Mean daily phosphorus (P) (± standard error) influent and effluent loads, mean daily P reduction, total P removal from respective supplemental treatments, combined supplemental treatment trains, and paired constructed floating wetland (CFW) + treatment train system per experiment (spring experiment = 133 days, fall experiment = 164 days). Order 1 sequence consists of an FeOx filter followed by a pine-bark bioreactor. Order 2 sequence consists of a pine-bark bioreactor followed by an FeOx filter

Experiment	Treatment order	Treatment technology (*n* = 12)	Mean daily influent P load (g·m^−3^·d^−1^)	Mean daily effluent P load (g·m^−3^·d^−1^)	Mean daily P reduction (g·m^−3^·d^−1^)	Mean supplemental P removed (g·m^−3^·d^−1^ )	Mean combined supplemental P removal (g·m^−3^·d^−1^)	Mean CFW + combined supplemental P removal (g·m^−3^·d^−1^)
Spring	Order 1	FeOx filter	0.26 ± 0.01	0.16 ± 0.01	0.10 ± 0.01	0.10 ± 0.01	0.13 ± 0.01	0.18 ± 0.01
		Bioreactor	0.16 ± 0.01	0.13 ± 0.01	0.03 ± 0.01	0.03 ± 0.01		
	Order 2	Bioreactor	0.27 ± 0.01	0.22 ± 0.01	0.05 ± 0.01	0.05 ± 0.01	0.14 ± 0.01	0.18 ± 0.01
		FeOx filter	0.22 ± 0.01	0.13 ± 0.01	0.09 ± 0.01	0.09 ± 0.01		
Fall	Order 1	FeOx filter	0.48 ± 0.01	0.25 ± 0.01	0.23 ± 0.01	0.23 ± 0.01	0.25 ± 0.01	0.24 ± 0.01
		Bioreactor	0.25 ± 0.01	0.23 ± 0.01	0.02 ± 0.01	0.02 ± 0.01		
	Order 2	Bioreactor	0.46 ± 0.01	0.43 ± 0.01	0.03 ± 0.01	0.03 ± 0.01	0.21 ± 0.01	0.23 ± 0.01
		FeOx filter	0.43 ± 0.01	0.26 ± 0.01	0.18 ± 0.01	0.18 ± 0.01		

The pine bark bioreactor containers were 37.9 L tubs (United Solutions Rough, Sardis, MS, USA) with a surface area of 0.17 m^2^ at the top and a volume of 0.07 m^3^. Each container was filled with 0.043 m^3^ of pine bark substrate (Timberline, Atlanta, GA, USA). Pine bark substrate was washed, oven-dried, and passed through sieves with pore diameters measuring 5.1, 3.8, 2.5, 1.9, and 1.3 cm before use. All particles > 5.1 cm and < 1.3 cm were discarded.

FeOx containers were 18.9 L buckets (Lowes, Mooresville, NC) with a top surface area of 0.113 m^2^ and a volume of 0.019 m^3^. The FeOx substrate used in this experiment was sieved to a size fraction of 0.5 to 4.0 mm for the spring experiment and 0.5 to 2.5 mm for the fall experiment. All FeOx media used in the current study were reclaimed from the passive treatment of net-alkaline coal mine drainage sludge at the Blue Valley Mine Drainage Treatment Station (Brandy Camp, PA, acquired through Iron Oxide Recovery Inc., Pittsburgh, PA). Blue Valley Iron Oxide (BVIO) is a commercially available and well-characterized source of FeOx that is produced from net-neutral mine impacted water, resulting in a higher purity FeOx compared to FeOx sourced from net-acidic coal mine drainage and additionally well below US Environmental Protection Agency limits for impurities for agricultural land application of sludges (Sibrell and Tucker 2012). For its major components, BVIO averages 68.4% Fe_2_O_3_, 0.53% Al_2_O_3_, 3.55% SiO_2_, and 2.71% CaO with 22.6% loss on ignition associated with hydrated compounds and volatilizable organics (Sibrell and Tucker 2012). Previous research from White et al. (2021) indicated an average sorption capacity of 18.3 ± 0.8 mg P·g FeOx^−1^ for this material. Using this sorption capacity and the predicted P load that the containers would receive over the course of each experiment, 2 kg of sieved FeOx was used in each container during each experiment.

### Phosphorus extraction from partially saturated FeOx

The dithionite-citrate-bicarbonate (DCB) technique was used to quantify the mass of P sorbed to the FeOx substrates. The DCB technique is typically used for extracting FeOx from soil material and plant roots and was adapted from Taylor and Crowder (1983). The DCB technique dissolves the FeOx and surface-adsorbed P into solution, allowing the P concentration within the resulting solution to be measured.

Following each experiment, water was drained from the FeOx containers, and a 2 cm diameter soil core was used to remove partially saturated FeOx samples for P extractions. One core sample was collected at the inlet where the PVC pipe delivered water to each container, followed by two samples at a random radial distance from the center in each of the four quadrants. Core samples were dried at 65 °C for 24 h. Extraction procedures were completed on the core sample from each container’s inlet and the samples collected from each of the four quadrants, totaling five samples per container. Dried FeOx from each sample was sieved to a size fraction of 0.5 to 2.0 mm, and 0.5 g of sieved FeOx was weighed out and added to 50 mL centrifuge tubes.

For the extraction procedure, 22.5 mL of 0.3 M sodium citrate solution and 2.5 mL of 1 M sodium bicarbonate solution were added to each tube. Tubes were placed in an 80 °C water bath with aluminum foil over the top to reduce evaporative loss. Once the solution reached 75 °C, 1 g of sodium dithionite was added, stirred for 1 min, and again for 1 min every 5 min, for a 15-min duration. After 15 min, 1 g of sodium dithionite was added to each tube, and the stirring procedure was repeated for ten more min. After 10 min, 1.5 mL of concentrated HCl was added to each tube to maintain a pH of < 4 in order to prevent re-adsorption and to maintain P in solution. Each tube was weighed to calculate evaporative losses and centrifuged at 1200 rpm at 25 °C for 15 min (Taylor and Crowder 1983). The supernatant was filtered through a 0.45 µm Luer lock filter. Following filtration, the supernatant of five samples (2.5 mL per sample) from each container was combined into a single composite sample (1 per treatment container) and stored at 4 °C until ICP-OES analysis. All ICP analyses had calibration standards rerun at the midpoint and end of each analytical run for quality assurance. Following extraction and analysis, concentrations of extracted P were reported in mg·L^−1^ and converted into mg P·g FeOx^−1^ using the volume of extracted supernatant.

### Data analysis

Nutrient concentrations determined by ICP analysis data were converted into total mass using container volume. Measured values below ICP detection limits were divided by two per Hewett and Ganser (2007), who state that if the percent of non-detectable (*D*_0_) values is less than 50% and the number of observations between 20 and 100, that substitution of D/2 is an appropriate approximation for statistical analyses. Container volume was corrected for rainfall additions and evapotranspiration to ensure that accurate inflow volumes were recorded to determine daily P loading rates and daily P load reduction following each treatment. Data for rainfall and evapotranspiration corrections were recorded to account for P dilution and concentration due to rainfall and/or evapotranspiration using an on-site Meter Group (Pullman, WA) weather station. Volumetric corrections were calculated by individual container area (m^3^) depending on rainfall and evapotranspiration, which ranged from 0.14 to 3.6 L·d^−1^.

Data were tested for distribution normality using the Shapiro–Wilk *W* test and homogeneity of variance using Levene’s test. Analysis of variance (ANOVA) was used to identify statistical significance (*p*-values < 0.05) after normality and independence assumptions were verified. When treatment effects were significant, a Student’s *t*-test was used to determine specific differences within the effects. All statistical calculations were performed using JMP® Pro 15.2.0 (SAS Institute Inc., Cary, NC).

## Results and discussion

### Effect of supplemental treatment order on P removal

**Fig. 3:**
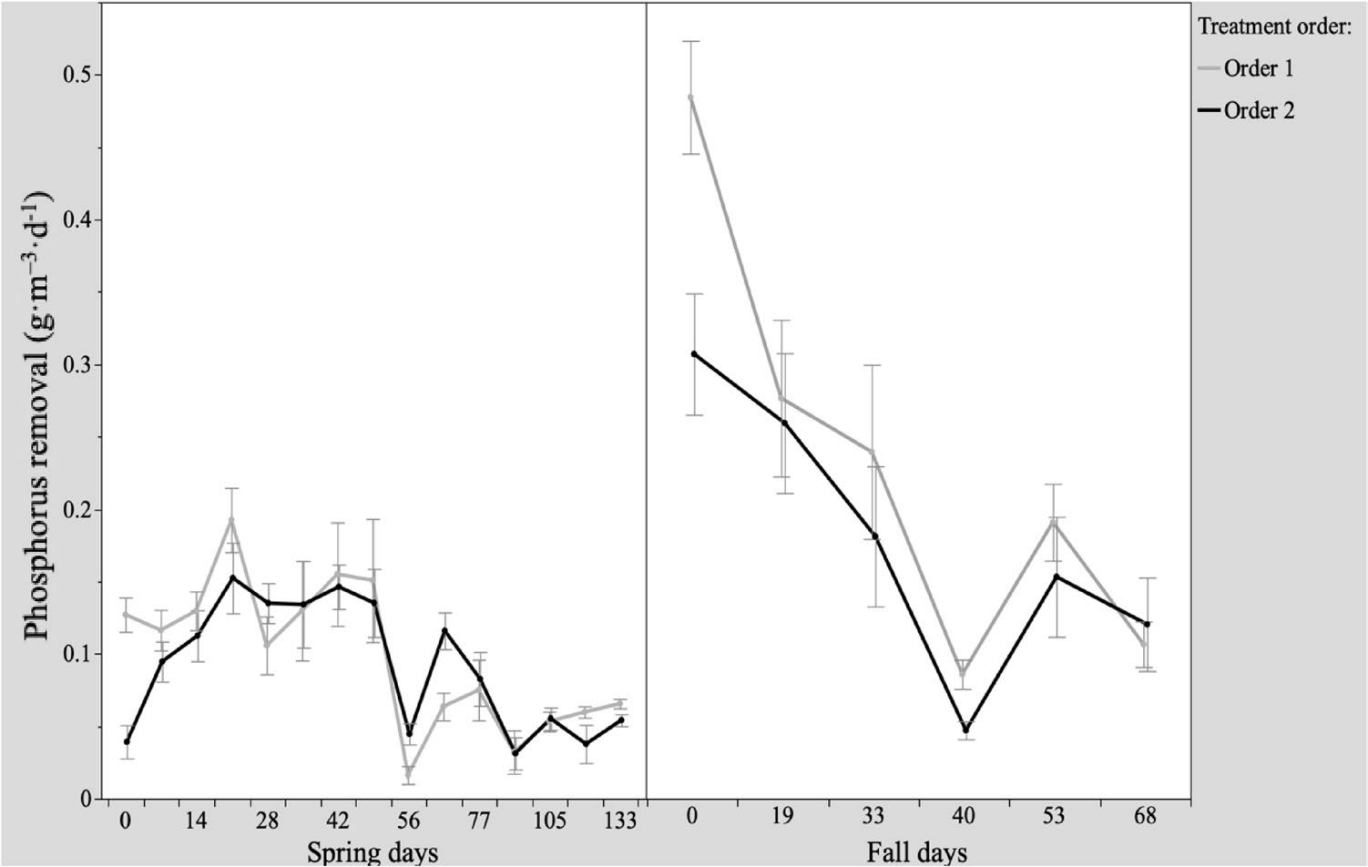
Mean daily phosphorus removal (± standard error) of Fe-Ox treatments by order (order 1: FeOx➔bioreactor; order 2: bioreactor➔Fe-Ox) in spring and fall experiments within FeOx treatments

During the spring experiment, the average daily P removal rate in the FeOx treatment containers did not differ when FeOx filters were placed upstream or downstream of the pine bark bioreactors (*p* = 0.57). However, the average daily P removal rate in the FeOx treatment containers during the fall experiment was higher when FeOx filters were placed upstream (order 1) of the pine bark bioreactors (*p* = 0.005). When assessing the P removal of the supplemental treatment train as a whole, across both experiments, P removal was marginally higher with FeOx treatments placed first (*p* = 0.11; Fig. 3). The most effective P removal placement was observed in order 1 during the fall experiment (*p* = 0.046; Fig. 3).

P removal in the pine bark bioreactors was lower than the FeOx treatments for both treatment orders (*p* < 0.0001). P was exported from pine bark bioreactors during select intervals of both experiments, but overall, a net positive P removal was attained (Table 1). While testing denitrifying woodchip bioreactors, Bell et al. (2015) reported an initial export of P followed by stabilization of P influent and effluent within 1 month of experiment initiation. Additionally, P removal has been reported by Choudhury et al. (2016), who determined that the high porosity and permeability of woodchip media contributed to particulate P reduction in denitrifying woodchip bioreactors. Christianson et al. (2017) determined that pollution swapping occurred with long bioreactor HRTs, which increased chemical oxygen demand and resulted in downstream FeOx (order 2) being the preferred order to polish bioreactor effluent. Theoretical HRTs in this study were 8.3 and 6.8 h in the spring and fall experiments, respectively; these HRTs did not negatively influence P removal as described by Christianson et al. (2017) and Goodwin et al. (2015).

### Conditions for P adsorption

Among the water quality parameters measured in this study, pH likely had the strongest influence on P adsorption to FeOx as the adsorption of P ions to the surface of FeOx is highly dependent on pH (Fish and Dietz 2009; Mao et al. 2011; Zhang et al. 2019). The surface charge of the FeOx substrate is influenced by both pH and the ionic strength of the water column, and previous research has indicated that P adsorption to FeOx generally decreases with increasing pH (Wilfert et al. 2015). According to Mao and Yue (2016), the P adsorption rate to hydrous FeOx decreased, while the desorption rate increased at higher pH levels (> pH of 8). Vicente et al. (2010) reported similar results, observing a 98% phosphate removal efficiency by iron particles at pH levels 5 and 6, 88% at pH 7, and 82% at pH 8–9. In the present study, the average pH across all experimental seasons and treatment orders ranged from 6.2 to 7.8; overall average pH across all treatments was 6.9, indicating an ideal setting for P sorption to FeOx (Table 2).

**Table 2:** Water quality parameters (± standard error) for FeOx filters following plant treatment during the two experimental periods

Experiment	Order	Parameter	Control	*Juncus effusus*	*Pontederia cordata*	Average
**Spring**	**1** FeOx → bioreactor (*n* = 60)	pH	7.6 ± 0.08	6.2 ± 0.19	6.6 ± 0.17	6.8 ± 0.10
		DO (mg/L)	10 ± 0.2	9.8 ± 0.33	10 ± 0.32	10 ± 0.17
		Temp (°C)	22 ± 0.89	21 ± 0.83	21 ± 0.84	21 ± 0.49
		ORP (mV)	178 ± 15	200 ± 15	180 ± 14	186 ± 8.5
	**2** Bioreactor → FeOx (*n* = 60)	pH	7.3 ± 0.06	6.6 ± 0.1	6.8 ± 0.09	6.9 ± 0.05
		DO (mg/L)	8.9 ± 0.38	9 ± 0.4	9.3 ± 0.44	9.1 ± 0.23
		Temp (°C)	22 ± 0.88	22 ± 0.86	22 ± 0.85	22 ± 0.50
		ORP (mV)	182 ± 14	182 ± 14	192 ± 14	186 ± 7.9
**Fall**	**1** FeOx → bioreactor (*n* = 48)	pH	7.8 ± 0.06	6.9 ± 0.09	7.3 ± 0.06	7.3 ± 0.05
		DO (mg/L)	8.2 ± 0.2	7.6 ± 0.2	7.6 ± 0.2	7.8 ± 0.12
		Temp (°C)	21 ± 0.88	19 ± 0.92	21 ± 0.82	20 ± 0.51
		ORP (mV)	371 ± 13	416 ± 14	378 ± 11	389 ± 7.35
	**2** Bioreactor → FeOx (*n* = 48)	pH	7 ± 0.05	6.7 ± 0.05	6.7 ± 0.04	6.8 ± 0.03
		DO (mg/L)	5.2 ± 0.24	5.6 ± 0.27	5.3 ± 0.31	5.4 ± 0.16
		Temp (°C)	19 ± 0.96	21 ± 0.87	20 ± 0.9	20 ± 0.52
		ORP (mV)	421 ± 14	412 ± 13	425 ± 14	419 ± 7.8

### Phosphorus content extracted from exposed FeOx

**Fig. 4:**
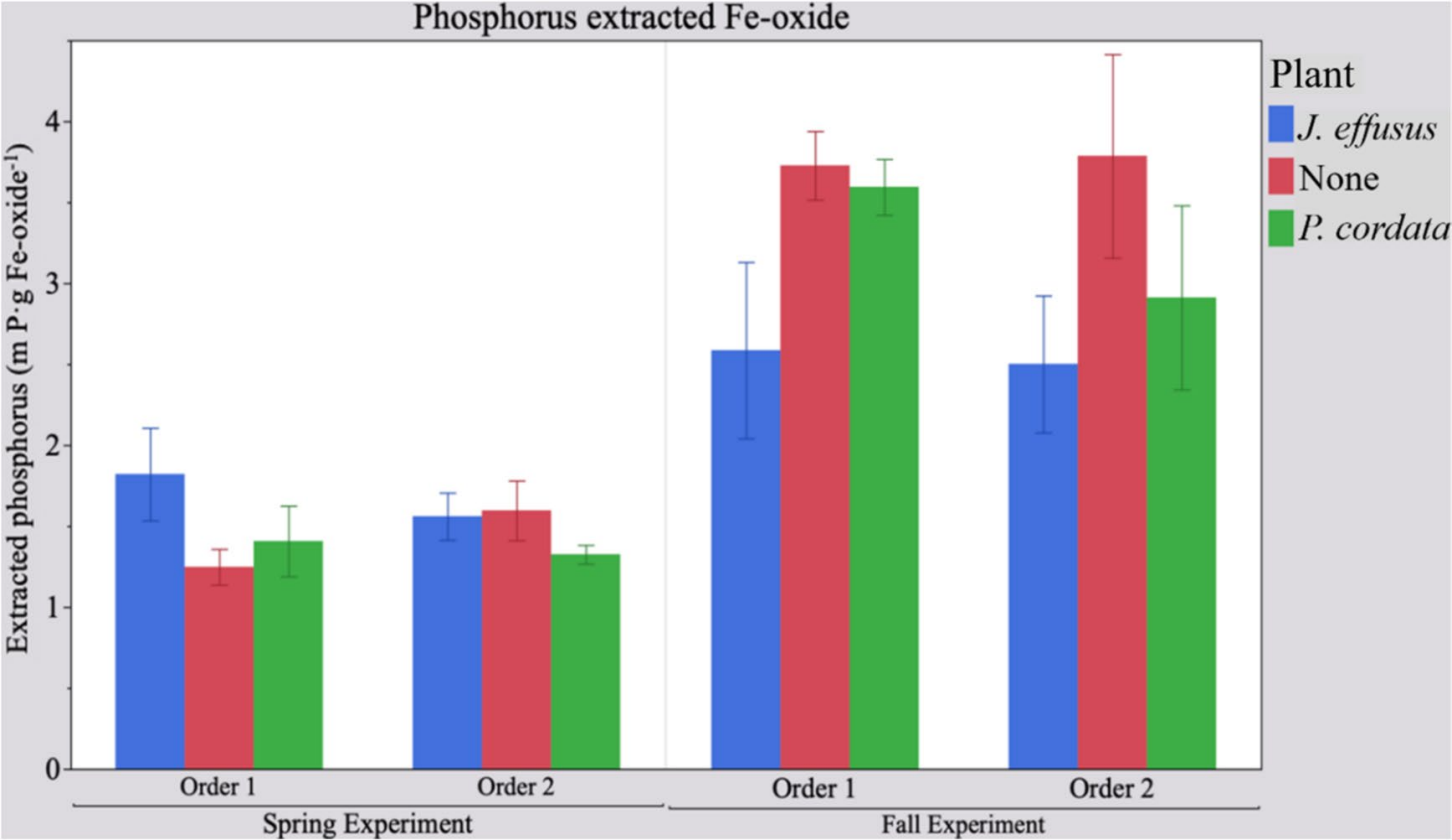
Mean concentration of P (± standard error) extracted from P-saturated Fe-oxides. Effluent from floating treatment wetland plant treatments flowed into supplemental treatments in two respective sequences (order 1: FeOx ➔bioreactor; order 2: bioreactor➔FeOx)

In comparing the amount P sorbed to FeOx between seasonal experiments, the P removed by FeOx filters after all planted treatment systems was higher in the fall experiment (Fig. 4; *p* ≤ 0.0001). Specific to the fall experiment, the amount of P sorbed to FeOx following the *J. effusus* treatment was lower than the amount of P sorbed to FeOx following *P. cordata* and non-vegetated control treatments (Fig. 4; *p* = 0.003). Because plant treatments were largely inactive during dormant winter months, the supplemental treatments received a higher P load as evidenced by the higher amount of P sorbed to the FeOx during the fall experiment.

When assessing the P removal capacity of the CFW and supplemental treatment trains in combination, more P was removed by the whole treatment train (CFW + FeOx filter + pine bark bioreactor) when effluent from the *J. effusus* treatment entered the supplemental treatments (*p* = 0.008). Effluent from the whole treatment system containing *J. effusus* had higher P removal, in part due to the higher nutrient uptake capacity of *J. effusus* plants in these experiments. Additionally, during the spring and fall experiments, the P removal capacity of the supplemental FeOx treatment alone was higher than the P removal of the CFWs (*p* < 0.0001). Figure 3 shows a sharp decline in the rate of P removal, particularly in the fall experiment, but this decline was likely due to partial saturation of P binding sites on the surface of the FeOx. Particularly in the fall experiment, these results indicate that the supplemental treatments received a higher P load due to diminished activity in the preceding plant treatments.

Although P removal by sorption was variable throughout both seasonal experiments, no P export from P previously sorbed to the FeOx filters was observed. The amount of P sorbed to FeOx used in this study (1.49 ± 0.08 mg P·g FeOx^−1^ and 3.18 ± 0.2 mg P·g FeOx^−1^ in the spring and fall experiments, respectively) is comparable to Wang et al. (2018), who similarly determined the amount of P sorbed to field-deployed sponge iron (0.5 mg P·g sponge iron). Other studies typically determined maximum sorption capacity, which assumes all P sorption sites were occupied on the substrate. Sorption sites on the FeOx deployed in the current study were not all occupied, which accounts for the lower amount of P sorbed (mg P·g FeOx^−1^) as compared to maximum sorption capacities from other studies in the literature (Table 3).

**Table 3:** Maximum sorption capacities of FeOx in the literature

Substrate description	Sorption capacity (mg P·g FeOx^−1^)	Source	Experiment type	Isotherm
Magnetic FeOx nanoparticles	5.03	Yoon et al. (2014)	Batch mode	Langmuir
Goethite (α-FeOOH)	6.7	Parfitt et al. (1975)	Batch mode	No isotherm modeling
< 0.15 mm FeOx in KH_2_PO_4_*	8.7	Sibrell et al. (2009)	Batch mode	Freundlich
Iron humate	10	Janoš et al. (2011)	Batch mode	Langmuir
FeOx tailings (30% FeOx)	12.6	Zeng et al. (2004)	Batch mode	Langmuir–Freundlich
Lepidocrocite (γ-FeOOH)	16.7	Parfitt et al. (1975)	Batch mode	No isotherm modeling
FeOx in irrigation reservoir water*	18.3	White et al. (2021)	Batch mode	Langmuir
Amorphous hydrous iron oxide	29.5	Parfitt et al. (1975)	Batch mode	No isotherm modeling
Granular FeOx (Bayoxide E33)	37.7	Lalley et al. (2016)	Batch mode	Langmuir

*Sourced from Blue Valley Iron Oxide

Although no desorption was observed in the P removal rates of FeOx filters, P release and subsequent P cycling were likely taking place in this experimental setup due to the long contact times of this flow-through system. This P release was evident in the CFW treatments that preceded the FeOx filters. Negative P removal values were likely due to nutrient release from senescing tissues within the CFW treatments, which contributed to a higher overall P load present in the experimental system (Dekle et al. 2024). Weng et al. (2012) reported that increases in dissolved organic carbon (DOC) within the water column can reduce phosphate sorption to substrate binding sites. Given that the FeOx filters used in this study received effluent from treatments containing aquatic plants (vegetated treatments) and algae (vegetated and unvegetated treatments), DOC levels were likely high compared to a system without preceding aquatic plants and algae. Additionally, short-circuiting likely occurred within the experimental system that decreased the hydraulic efficiency and contributed to lower actual HRTs within the system (Persson et al. 1999).

The cited studies in Table 3 were completed using batch systems as opposed to flow-through, as in this study. Since there was only one FeOx sample interval per season in this study, future studies would benefit from sampling FeOx at specific time intervals throughout the study to quantify any sorption and/or desorption dynamics occurring over time within a flow-through system. Future studies should also quantify DOC concentrations throughout the treatment cells to better understand the role of DOC in P sorption dynamics in these novel systems. The use of P sorption substrates has the potential to enhance overall P removal when used in combination with constructed wetland technologies. Research by Wang et al. (2018) reported enhanced P removal using CFWs modified with zeolite or sponge iron substrates suspended in the water column. Previous research by White et al. (2021) demonstrated the potential for P-saturated FeOx to be used as a soil amendment, where slow desorption of P from the surface of saturated FeOx provided adequate plant-available P.

## Conclusions

FeOx can facilitate P removal in high P-loaded systems when plant nutrient uptake is limited during plant dormancy. The P removal rate of FeOx filters (0.095–0.23 mg·m^−3^·d^−1^) was higher than that achieved by the preceding CFW treatments (− 0.014–0.056 mg P·g FeOx^−1^). The order of supplemental treatments influences P removal when plant treatments are less active in fall and winter months. However, treatment order does not influence P removal rates in spring and summer months when plants within CFWs are actively growing. In combination with plant-based treatment technologies, FeOx filters receiving effluent directly from CFW treatments removed 33–50% of the P load.

Reclaimed FeOx filters are a cost-effective sorption substrate for reducing P loads present in nutrient-enriched systems. It is unlikely that sorption substrates will reach maximum sorption capacity in field settings because of the non-ideal flow paths and heterogeneity of larger-scale systems. Future studies could benefit from continuous, year-round monitoring, and sampling of FeOx treatments to determine field-specific P-saturation indices for FeOx.

Although FeOx filters are effective as a P treatment strategy, maintenance (periodic substrate mixing, removal, and replacement once FeOx nears saturation) is required to maintain continuous functionality. Further research is needed to focus on the combination of treatment technologies to better characterize the effectiveness of sorption-based substrates and their combination with other nutrient removal mechanisms. Future FeOx filter designs should focus on maximizing substrate contact time, enhancing the scalability of P treatment capacity across a variety of industries.

Implementing FeOx-based remediation at an operational scale will aid agricultural operations, stormwater managers, and water treatment facilities in reducing nutrient contamination for improving on and off-site water quality. This research is aimed at better understanding nutrient dynamics within two-stage supplemental treatments to inform design of low-cost treatment systems. Installation of treatment trains could assist operations focused on P removal and P recycling. Overall, these experiments show that FeOx filters can serve as an effective substrate for P remediation to supplement other nutrient remediation technologies that are limited by seasonality.

## Data Availability

All relevant data are included in the paper. Raw data are available via Creative Commons Licence 4.0 – and open access – FigShare https://doi.org/10.6084/m9.figshare.26081893.v1.
